# Sarcoidosis in gastric cancer at the time of diagnosis: A case report

**DOI:** 10.3892/ol.2015.2850

**Published:** 2015-01-05

**Authors:** YANG JIAO, JIE NING, WEN-DI ZHAO, YAN-LI LI, HONG-YANG WU, KANG-SHENG GU

**Affiliations:** 1 Department of Oncology, The First Affiliated Hospital of Anhui Medical University, Hefei, Anhui 230022, P.R. China; 2 Department of Pathology, The First Affiliated Hospital of Anhui Medical University, Hefei, Anhui 230022, P.R. China; 3 Department of Hematology, The Second Affiliated Hospital of Anhui Medical University, Hefei, Anhui 230601, P.R. China

**Keywords:** sarcoidosis, gastric cancer, diagnosis, pathology

## Abstract

Sarcoidosis is a multisystemic inflammatory disease that commonly affects the lungs and lymphatic system and is characterized by the formation of non-caseating granulomas. Although the association between sarcoidosis and malignant diseases has been well described, it remains controversial whether this association is merely a coincidence or the consequence of a common pathophysiological mechanism. The present study reports a rare case of sarcoidosis that was present in a patient with gastric cancer at the time of diagnosis. A 64-year-old female diagnosed with stage I gastric cancer underwent curative surgery, and the postoperative pathology of the lymph nodes revealed non-caseating granulomas. At the 4-year follow-up, the sarcoidosis remained stable, and no recurrence of cancer was identified. The present case revealed that sarcoidosis and gastric cancer may coexist simultaneously and focused on the potential advantages of histological confirmation in patients with cancer and sarcoidosis.

## Introduction

Sarcoidosis is a systemic disorder of unknown etiology that is characterized by the widespread development of non-caseating epithelioid cell granulomas in multiple organ systems (1). The widely accepted pathogenic hypothesis is that sarcoidosis is promoted in genetically susceptible individuals by environmental factors (2). Non-caseating granulomas consisting of epithelioid cells and T lymphocytes are the characteristic lesions of sarcoidosis (2). The association between sarcoidosis and malignancy has been reported by several studies with conflicting results; this is caused by the clinical and radiographic features of the disease, which exhibit similarities to malignancies such as lymphoma or lung cancer and were occasionally in the absence of histological confirmation (3,4). The present study reports a case of sarcoidosis mimicking cancer metastasis that was present at the time of the diagnosis of gastric cancer and discusses the diagnostic process. The patient provided written informed consent.

## Case report

**Figure 1:**
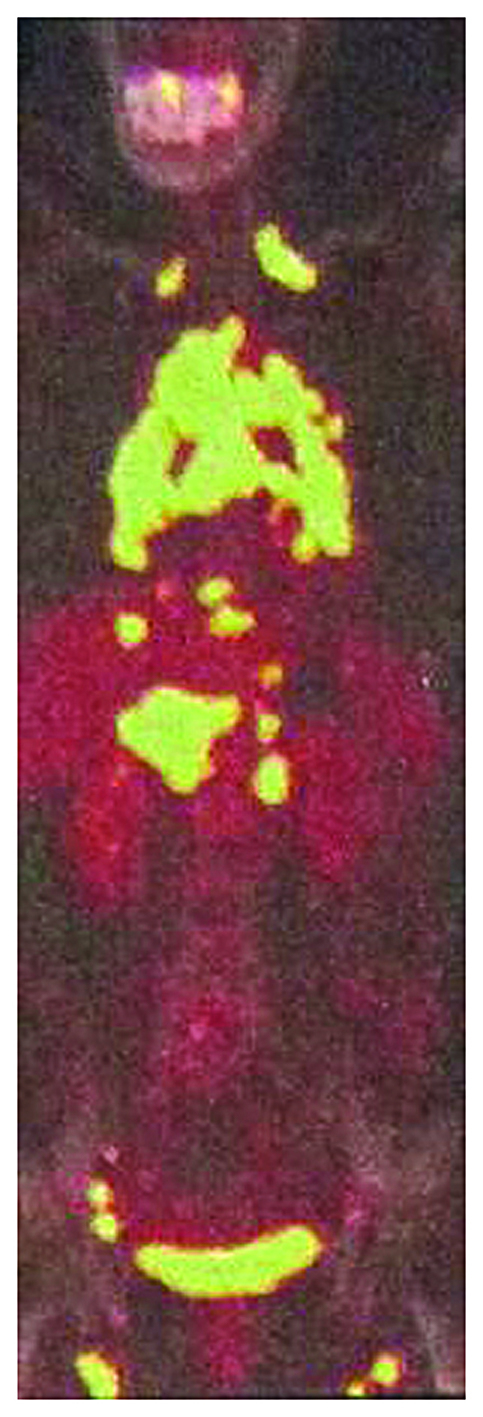
PET/CT images of the lymph nodes. A PET/CT scan revealing multiple areas of high fluorodeoxyglucose uptake in the bilateral supraclavicular, mediastinal, hilar, retroperitoneal, pelvic and inguinal lymph nodes. PET/CT, positron emission tomography and computed tomography.

**Figure 2:**
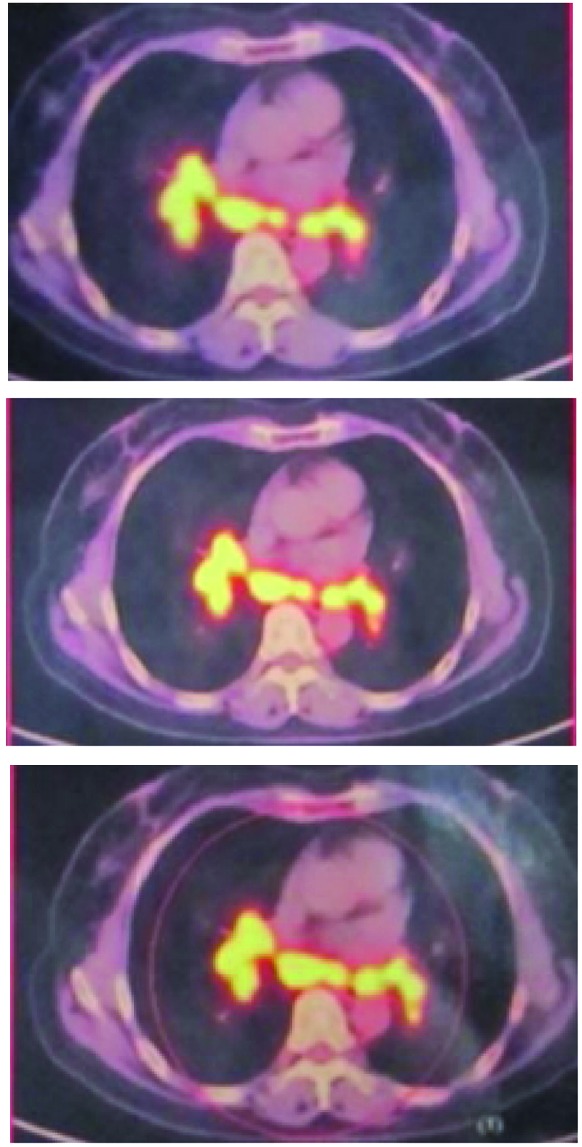
Positron emission tomography and computed tomography images of the mediastinal and hilar lymph nodes.

A 64-year-old female presented to the Lujiang County Hospital (Hefei, China) with epigastric discomfort and a swallowing disorder that had persisted for more than one month. During the course of the disease, the symptoms of night sweating and weight loss were also present, however, no fever, coughing, expectoration, hemoptysis, nasal congestion, hematemesis or melena occurred. The patient had previously suffered from esophageal erosion. Gastroscopy revealed a gastric lesion that was located in the cardia, and pathological analysis of the lesion revealed high-grade intraepithelial neoplasia and local canceration. Bilateral hilar widening and enlarging of multiple lymph nodes in the mediastinum and hilum of the lung were revealed by chest X-ray and chest-abdomen computed tomography (CT), respectively. Positron emission tomography (PET)/CT revealed that the fluorodeoxyglucose metabolism of the lymph nodes in the bilateral supraclavicular, mediastinal, hilar, retroperitoneal, pelvic and inguinal regions had increased (Figs. 1 and 2). Based on these results, two types of disease were considered, lymphoma and a rare digestive tract tumor with multiple metastases. Lujiang County Hospital diagnosed the lesion as advanced gastric cancer.

**Figure 3:**
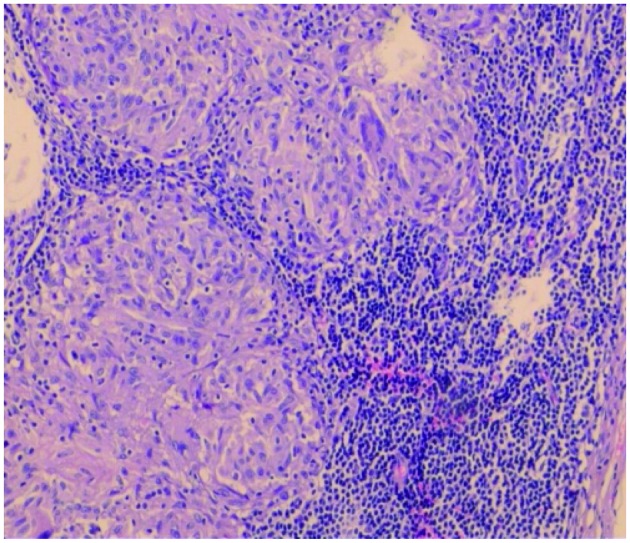
High-power photomicrograph of the biopsy specimen from the right inguinal lymph node revealing granulomatous infiltration with occasional multinucleated giant cells (hematoxylin and eosin stain; magnification, ×100).

In order to confirm the diagnosis and undergo treatment, the patient presented to the outpatient department of the First Affiliated Hospital of Anhui Medical University (Hefei, China). Upon physical examination, a tenacious lymph node, ~1.0×1.0 cm in size, was identified in the left supraclavicular root of the neck and several lymph nodes in the right inguinal region. No other lymph node swelling was observed in the superficial part of the body. Laboratory investigation indicated that a routine blood examination (white blood cells, 4.8×10^9^ cells/l, normal range, 4–10×10^9^ cells/l; hemoglobin, 115 g/l, normal range, 110–150 g/l; platelet count, 224×10^9^ cells/l, normal range, 100–300×10^9^ cells/l), liver (aspartate transaminase, 20 IU/l, normal range, 0–40 IU/l; alanine transaminase, 22 IU/l, normal range, 0–40 IU/l) and kidney function (blood urea nitrogen, 4.9 mmol/l, normal range, 2.1–7.9 mmol/l; creatinine 85μmol/l, normal range, 44–133 μmol/l) tests were normal, the serum calcium level was 2.75 mmol/l (normal range, 2.25–2.75 mmol/l) and the alkaline phosphatase level was 135 U/l (normal range, 25–125 U/l). Fine needle aspiration biopsy was performed on the left supraclavicular lymph node, and revealed lymphoreticular hyperplasia, as epithelioid cells were present. Due to the cytology results, a tuberculosis examination was performed, but the tuberculin testing exhibited a negative result. The biopsy of the right inguinal lymph node indicated granulomatous infiltration with occasional multinucleated giant cells (Fig. 3).

**Figure 4:**
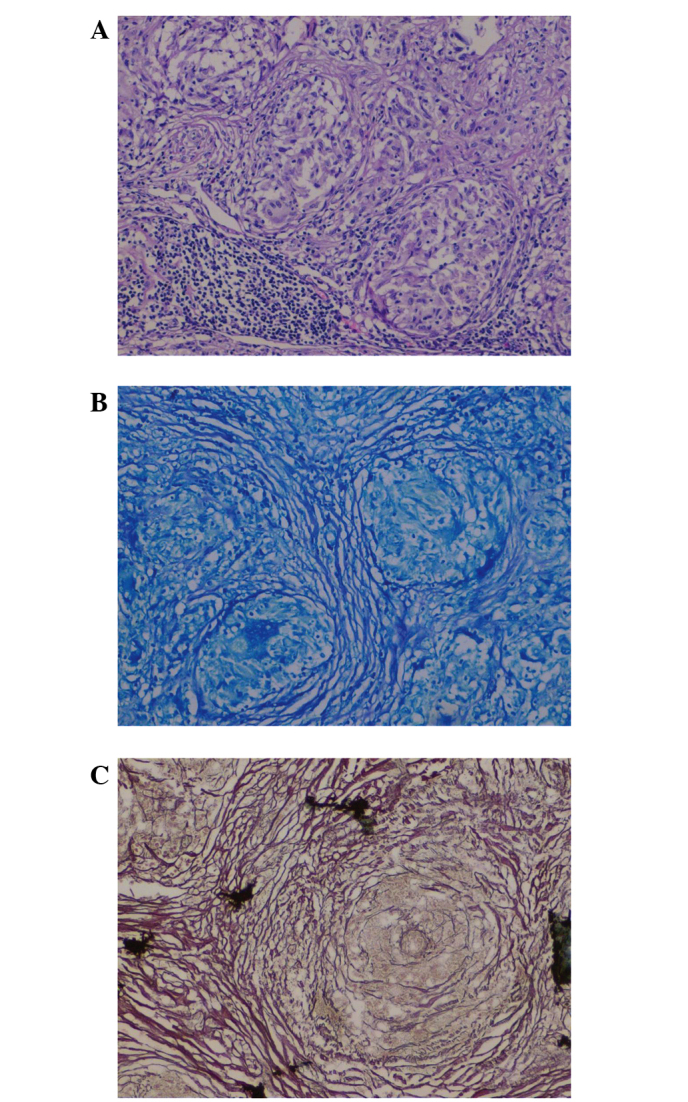
High-power photomicrograph of the biopsy specimen from the gastric lymph node revealing non-caseating granuloma. (A) Hematoxylin and eosin stain; magnification, ×100, of the non-caseating granulomatous lesions. (B) Acid fast stain of the lesions revealing non-acid fast cells. (C) Reticular fiber-specific staining revealing numerous reticular fibers inside and around the granulomatous lesions.

The patient was referred to the Department of General Surgery at the first Affiliated Hospital of Anhui Medical University, having provided the appropriate informed consent, and received radical total gastrectomy combined with a Roux-en-Y procedure (5). A superficial ulcerative mass was identified inside the small curved side below the cardia and multiple enlarged lymph nodes were identified around the stomach and liver duodenum ligament, the largest of which was 2.5×2.0 cm in diameter. The postoperative pathology indicated erosive medium differentiated adenocarcinoma in the lesser gastric curvature, ~2.0×1.5 cm in diameter. The cancer cells had invaded the layer of muscularis mucosae and a number of cells had reached the submucosa. In total, eight lymph nodes were dissected from the lesser curvature, one from the greater curvature and 23 from other areas surrounding the gastric region. The pathology of the total 32 lymph nodes indicated the presence of non-caseating granulomas (Fig. 4A and B). Therefore, the patient was diagnosed with gastric erosive medium-differentiated adenocarcinoma (pT1N0M0) (6) and sarcoidosis (I) (1). According to the NCCN guidelines for gastric cancer staging result determination, the patient did not receive chemotherapy (7). At the four-year checkup, the sarcoidosis remained stable, and no recurrence of the cancer was identified.

## Discussion

The present study reports a rare case of sarcoidosis that was present in a patient with gastric cancer at the time of diagnosis, which highlights the complexity of the diagnostic process. The present case identifies that clinical presentation and radiological findings, including PET/CT, may not be able to differentiate between cancer metastasis and sarcoidosis.

Sarcoidosis has been reported to imitate malignant neoplasms and has been identified at the time of diagnosis and during or immediately following chemotherapy, including in cases of breast (8–10) and lung (11–13) cancer, Hodgkin’s disease (14,15), colorectal (16–18) and head and neck cancer (19,20), melanoma (21–23) and ovarian cancer (24). Although cases of sarcoidosis mimicking metastatic gastric cancer have been rarely reported, Konishi *et al* (25) reported a case of advanced gastric cancer with sarcoidosis and Blank *et al* (26) revealed that four patients with gastric cancer were identified in their cohort of 425 patients with sarcoidosis.

The association between sarcoidosis and malignancy remains controversial. Brincker and Wilbek (27) first reported a statistically significant increase in the incidence of malignant tumors among patients with sarcoidosis in 1974. A large Japanese study followed 1,411 patients with sarcoidosis for three years and also observed an increase in mortality from leukemia and uterine cancer, using a standardized mortality risk (28). A retrospective cohort study by Askling *et al* (29) analyzed two cohorts of patients with sarcoidosis and identified an increased risk of lymphoma and lung, liver, and skin cancer. Boffetta *et al* (30) reported that the risks of rectal, colon and kidney cancer were increased in patients with sarcoidosis in their cohort study. In addition, 1,045 out of a total of 10,037 hospitalized patients with sarcoidosis were reported to subsequently develop cancer, with a significant proportion being reported for skin (squamous cell), kidney and non-thyroid endocrine tumors and additionally for non-Hodgkin’s lymphoma and leukemia (31). Furthermore, in the Medical Center of the University of Heidelberg (Heidelberg, Germany), 61 patients with malignant disease were identified in the cohort of 425 patients with sarcoidosis (26). However, other studies have hypothesized that malignancy may actually precede the diagnosis of sarcoidosis. Suen *et al* (32) reported six cases in which sarcoidosis was diagnosed an average of nine months following the development of malignancy and termed this phenomenon malignancy-sarcoidosis syndrome. Despite the evidence suggesting that these two entities may be linked, a number of studies have challenged the existence of an association between sarcoidosis and malignancy. Rømer *et al* (33) reviewed the cases of sarcoidosis and malignancy that Brincker and Wilbek (27) had presented, finding that a number of cases had been misclassified and identified no increased occurrence of malignancy in patients with sarcoidosis. The importance of misclassification was also reported by Seersholm *et al* (34), who identified misclassification in three out of 36 malignancies in 254 patients with sarcoidosis.

Conflicting results among these various studies are caused by the similarities in the clinical and radiological features of sarcoidosis and malignancy and occasionally, the lack of histological confirmation. PET/CT imaging is a valuable tool for the diagnosis of cancer and for monitoring cancer metastasis as it allows for the location of metabolically active malignant tissue to be determined. However, the specificity of PET/CT is hampered by non-oncological medical conditions, including sarcoidosis, Wegener’s granulomatosis, chronic granulomatous disease, and mycobacterial and aspergillus infections, where glucose consumption may be observed, particularly in the cellular component of inflammatory lesions. Therefore, it is important to obtain a histological diagnosis prior to initiating antineoplastic therapy based on the imaging findings.

Sarcoidosis must be considered in differential diagnosis when a cancer patient develops diffuse lymph nodal involvement. As the currently available imaging techniques fail to formally distinguish metastasis from sarcoidosis, a pathological diagnosis must be obtained wherever possible.
